# Robust Strain Sensor with High Sensitivity Based on Polymer-Encapsulated Microfiber Mach–Zehnder Interferometer

**DOI:** 10.3390/polym16192810

**Published:** 2024-10-03

**Authors:** Bin Xiao, Funa Zhuang, Jing Wang, Zhongyu Yao, Shanshan Wang

**Affiliations:** Optics and Optoelectronics Laboratory, College of Physics and Optoelectronic Engineering, Ocean University of China, Qingdao 266100, China; xiaobin@stu.ouc.edu.cn (B.X.); zhuangfuna@stu.ouc.edu.cn (F.Z.); wjing@ouc.edu.cn (J.W.); yaozhongyu1102@163.com (Z.Y.)

**Keywords:** microfiber Mach–Zehnder interferometer, polymer, strain sensor

## Abstract

A robust strain sensor is demonstrated based on a microfiber Mach–Zehnder interferometer (MMZI) encapsulated by the polymer polydimethylsiloxane (PDMS). Benefiting from the low Young’s modulus of PDMS, both a robust structure and high sensitivity can be realized based on three different encapsulations. In the experiment, the proposed sensors are fabricated and tested with strain sensitivities ranging from −20.95 pm/με to 127.00 pm/με within the wavelength range of 1200–1650 nm. Compared with the bare MMZI sensor, at least one order of magnitude higher sensitivity is reached. To further evaluate the performance of the sensor, the dependences of sensitivity on probing wavelength and the different types and quantities of polymers used in encapsulation are discussed. Results show that the sensitivity of the sensor will increase with the probing wavelength. The type and quantity of polymer used are also very critical to sensitivity. Additionally, a response time of 24.72 ms can be reached. Good recoverability and repeatability of the sensor are also demonstrated by repeated experiments. The strain sensor demonstrated here shows the advantages of simple fabrication, robust structure, high and tunable sensitivity, fast response, good recoverability and repeatability.

## 1. Introduction

Strain sensors are important for the structure monitoring of large buildings, oil and gas pipelines [1,2,3], earthquakes [4] and medicine areas [5]. In these applications, strain can be used as an important indicator to help people judge the aging of buildings, corrosion of pipelines, damage to ships, severity of earthquakes and degree of human health. In order to realize strain measurement, many different types of sensors have been developed, such as resistive strain sensors [6], capacitive strain sensors [7], non-contact strain sensors [8] and sonic strain sensors [9]. However, these strain sensors usually suffer from the problems of complicated preparation methods, difficult operation, being easy to corrode and environment pollution. In contrast, fiber-optic sensors have attracted more and more research interest due to their advantages of anti-electromagnetic interference, low cost and easy operation.

In recent years, many kinds of fiber-optic sensors for strain sensing and measurement have been proposed and demonstrated, including sensors based on fiber Bragg grating (FBG) [10,11,12], long-period fiber grating [13,14], Mach–Zehnder interferometer (MZIs) [15,16,17,18,19,20], Fabry–Perot interferometer (FPIs) [21,22], distributed fiber-optic sensors [23] and hybrid structures [24], in which the interferometric fiber sensors including open-cavity interferometers and S-tapered fiber MZIs show relatively high sensitivity. For these kinds of sensors, benefiting from a tapered fiber, strain sensitivity is improved significantly. However, they also suffer from the problem of robustness simultaneously. After all, strain sensors have to bear the stress induced by ambient objects or surroundings.

Besides the above-mentioned open-cavity interferometer or S-tapered fiber MZI, interferometric sensors fabricated by microfiber have also been developed for strain sensing and measurement, such as sensors based on a dual-arm MZI [25], in-line MZI [15] and microfiber Mach–Zehnder interferometer (MMZI) [26]. In particular, the MMZI sensors have received more and more attention due to their advantages of easy fabrication, high sensitivity and fast response. Similarly, robustness is still a challenge for this kind of structure due to the thinner fiber used. In other words, it will be a very meaningful thing to develop a strain sensor that is robust and highly sensitive for practical applications.

To develop a robust strain sensor with high sensitivity, a polymer material with a low Young’s modulus (such as polydimethylsiloxane (PDMS) with a Young’s modulus of 7.5 × 10^5^ Pa) is combined with a microfiber sensor, such as an MMZI. Benefiting from the PDMS encapsulation, both the robustness and sensitivity of the MMZI are improved significantly. In our experiment, an MMZI is fabricated and encapsulated with three different methods. In particular, some metal baffles are introduced to control the amount of PDMS used in encapsulation. The measured strain sensitivity ranges from −20.95 pm/με to 127.00 pm/με, which is one or two orders of magnitude higher than that of an MMZI without encapsulation. To further investigate the performance of the sensor, the effects of probing wavelength and the type and amount of polymer used in encapsulation on sensitivity are also discussed. In addition, a fast response, good recoverability and repeatability of the sensor are also demonstrated experimentally. The sensors demonstrated here can balance robustness and high sensitivity well, which has potential in the applications of the health monitoring of small building structures, pipeline transportation and so on.

## 2. Theory and Sensor Fabrication

### 2.1. Theory

The sensing theory of the MMZI strain sensor can be described as follows: higher-order modes (such as the *HE*_12_ mode) are excited in the transition region of an MMZI produced by the non-adiabatically tapering process. Meanwhile, phase accumulation of all modes (including the fundamental mode and high-order modes) begins. When they meet at another transition region of the fiber, an interference spectrum between the fundamental mode and high-order modes is formed.

Based on two-beam interference theory, the light intensity of the transmission spectrum generated can be expressed as follows:

(1) $$I=I_{1}+I_{2}+2\sqrt{I_{1}I_{2}}\cos\Delta \varphi ,$$

where *I* is the total intensity of the spectrum, *I*_1_ is the intensity of the core mode and *I*_2_ is the intensity of the cladding mode (high-order mode). Δ*φ* is the phase difference between these two modes, which can be expressed as

(2) $$\Delta \varphi =\frac{2\pi \Delta n_{eff}L}{\lambda },$$

where Δ*n*_eff_ is the effective refractive index (ERI) difference between the core and cladding mode. *L* is the length of the interferometer and *λ* is the wavelength of the probing light. When the phase difference Δ*φ* satisfies Δ*φ* = (2*m* + 1) π (*m* is an integer), the total intensity *I* reaches the minimum value, which corresponds to the dip of the spectrum. The wavelength of the dip *λ_m_* can be expressed by

(3) $$\lambda_{m}=\frac{\Delta n_{eff}L}{2m+1}.$$

The distance between two adjacent attenuation dips, known as the free spectral range (FSR), can be expressed as follows:

(4) $$FSR=\frac{\lambda_{m}^{2}}{\Delta n_{eff}L}.$$

When external stress is applied to the MMZI without encapsulation, because of the photo-elastic effect, the refractive index of the core and cladding will be changed. As a result, the Δ*n_eff_* and the phase difference between the core and cladding modes will also be changed. The changed phase difference causes a dip shift in the transmission spectrum, which can be expressed by [27]

(5) $$\Delta \lambda_{m}=(1+\frac{P_{e1}n_{1}-P_{e2}n_{2}}{n_{1}-n_{2}})\lambda_{m}\Delta \varepsilon ,$$

where *n*_1_, *n*_2_ and *P*_e1_, *P*_e2_ are the ERI and elasticity coefficient of two modes, respectively, and Δ*ε* is the change in strain. Based on Equation (5), the following expression can be obtained:

(6) $$S_{\varepsilon }=\frac{\Delta \lambda_{m}}{\Delta \varepsilon }=(1+\frac{P_{e1}n_{1}-P_{e2}n_{2}}{n_{1}-n_{2}})\lambda_{m}=(1+P_{e})\lambda_{m},$$

where *P*_e_ is a constant related to the material parameters. *S_ε_* is the strain sensitivity of the sensor. When the MMZI is encapsulated by a polymer, the *P*_e_ increases significantly and different encapsulations will lead to different *P*_e_.

### 2.2. Sensor Fabrication

**Figure 1:**
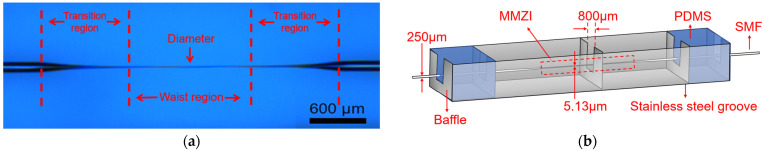
(**a**) Microscope image of the fabricated MMZI. (**b**) Schematic diagram of encapsulation.

The MMZI strain sensor is fabricated by the two-step stretching method with single-mode fiber (SMF). This method has been used and described in other references [28,29]. An optical microscope photo of the fabricated MMZI is shown in Figure 1a. The diameter of the waist region is measured as 5.13 μm, the length of the waist region as 1290.91 μm and the length of the transition regions as 945.30 μm and 982.16 μm, respectively. Based on the fabricated MMZI, the MMZI sensor is encapsulated by a polymer. As revealed in Section 2.1, the introduction of the polymer around the MMZI sensor can help to improve the sensitivity and robustness. After all, during the strain sensing process, the sensor will inevitably bear stress.

In encapsulation, PDMS is used, which is mainly due to its low Young’s modulus and moderate refractive index. Specifically, PDMS shows a low Young’s modulus and strong elasticity. It is known that the lower the Young’s modulus of the material, the poorer the ability to resist the deformation. Therefore, the PDMS encapsulation can improve the strain sensitivity of the MMZI and ensure its stability simultaneously. In addition, the refractive index of PDMS (*n* = 1.408) is also moderate.

The encapsulation process can be described as follows: Firstly, the fabricated MMZI is lowered to a stainless steel groove with the help of a displacement table. Then, Reagent A (the main reagent, model 2184, Hamld) and Reagent B (curing reagent) of PDMS are mixed in a proportion of 10:1. Afterwards, the mixed PDMS liquid is dropped into the stainless steel groove and heated under 110 °C for 20 min until it solidifies with the help of a heating platform with temperature control function. In particular, to control the amount of the polymer used accurately, some baffles made of metal sheets are placed before the PDMS is dropped, by which the polymer is confined to a limited space. A schematic diagram of the encapsulated MMZI is shown in Figure 1b.

**Figure 2:**
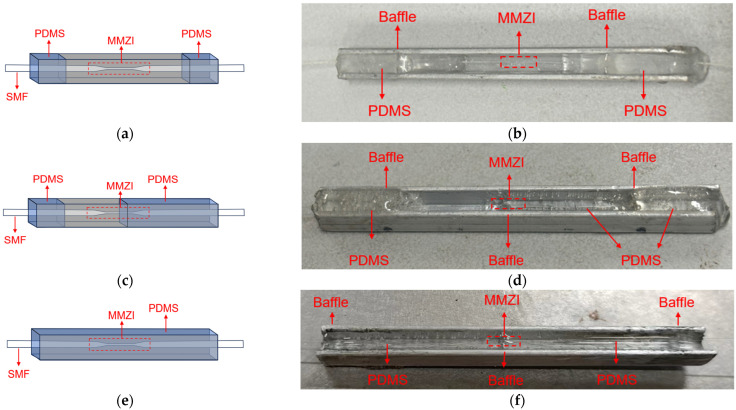
(**a**) Schematic diagram and (**b**) photo of MMZI with two-end encapsulation. (**c**) Schematic diagram and (**d**) photo of MMZI with semi-encapsulation. (**e**) Schematic diagram and (**f**) photo of MMZI with full encapsulation.

In addition, in order to investigate the effect of different encapsulations on strain sensing, MMZI sensors with different encapsulations are designed. According to different exposures of the waist region, they are classified into three types: two-end encapsulation, semi-encapsulation and full encapsulation, as shown in Figure 2. The two-end encapsulation means that the drop-added PDMS exists only at the two ends of the optical fiber, and the waist region of the optical fiber is fully exposed to the air. Semi-encapsulation means that half of the MMZI is immersed into the PDMS and full encapsulation indicates that the whole MMZI sensor is covered by the PDMS.

The encapsulation method is similar to that described above. However, for different encapsulations, the baffles are placed at different positions to control the different amount of polymer used in encapsulation. The photos of the MMZI sensors with different encapsulations are shown in Figure 2b, Figure 2d, and Figure 2f, respectively.

## 3. Experimental Results

**Figure 3:**
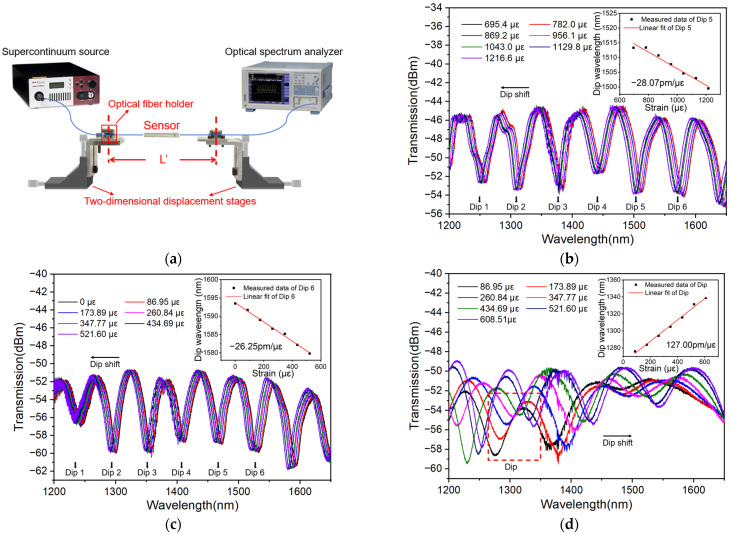
(**a**) Schematic diagram of test system. Transmission spectra of MMZI with (**b**) two-end encapsulation, (**c**) semi-encapsulation and (**d**) full encapsulation.

To test the strain response of the sensor, a test system is established as follows: two-dimensional displacement stages are used to fix and stretch the two ends of the MMZI. One end of the MMZI is connected to a supercontinuum light source (NKT Photonics, SuperK Compact, Birkerød, Denmark) for light launching and the other end of the MMZI is connected to an optical spectrum analyzer (OSA, Yokogawa AQ6370C, Tokyo, Japan) for signal collection, as shown in Figure 3a. The light source is a kind of white light with a wavelength range of around 450–2400 nm. In the experiment, only the spectrum within the wavelength range of 1200–1650 nm is tracked, which is mainly because the longer the wavelength used, the higher sensitivity that can be reached. On the other hand, the upper limit of the wavelength range is limited by the detection range of the OSA used.

In the experiment, by turning the actuator of the displacement stage, axial stress is applied to the sensor. Once the actuator turns one round, a moving distance of 0.01 mm is created in the axial direction. Combining the length *L′* between two fixed points as shown in Figure 3a, the strain *ε* applied to the sensor can be evaluated by the following expression:

(7) $$\varepsilon =\ln\frac{L^{\prime }+\Delta L^{\prime }}{L^{\prime }}.$$

where ∆*L′* indicates the displacement change of the stage. Sensing experiments on the MMZI sensors with different encapsulations are performed based on the established system. Results are shown in Figure 3b–d, respectively. Evidently, different sensors show different strain responses. By tracking and linear fitting of typical dips in transmission spectra, typical sensitivities of −28.07 pm/με (central wavelength 1507.46 nm), −26.25 pm/με (central wavelength 1586.86 nm) and 127.00 pm/με are obtained for MMZI sensors with two-end encapsulation, semi-encapsulation and full encapsulation, respectively. Compared with the MMZI without encapsulation, at least one order of magnitude higher sensitivity is reached [26].

The reason why the trend in dip wavelength versus the wavelength for full encapsulation is completely different from the two-end capsulation and semi-encapsulation is mainly due to the different materials around the waist region of the MMZI. Specifically speaking, for the semi-encapsulation sensor, the waist region of the MMZI is surrounded by air. However, for full encapsulation, the waist region of the MMZI is surrounded by PDMS. In addition to Equation (6), the strain sensitivity of the sensor can be expressed as follows [28]:

(8) $$S_{\varepsilon }=\frac{d\lambda }{d\varepsilon }=\frac{1}{\frac{\Delta n_{eff}}{\lambda }-\frac{\partial (\Delta n_{eff})}{\partial \lambda }}.\frac{\partial (\Delta n_{eff})}{\partial \varepsilon }.$$

It is known that the *n_eff_* is dependent on the fiber diameter, probing wavelength and surrounding. When the surrounding is changed from air to PDMS, due to the large ERI difference between air and PDMS, the *n_eff_* and Δ*n_eff_* are also changed significantly. As a result, all the dependences will be changed. Especially, when the symbol of the difference between $\frac{\Delta n_{eff}}{\lambda }$ and $\frac{\partial (\Delta n_{eff})}{\partial \lambda }$ is changed, the sign of sensitivity will also be changed.

In addition, almost one order of increment in the sensitivity of our sensor is observed, which mainly benefits from the large FSR of the sensor fabricated. Usually, the larger the FSR is, the higher sensitivity that can be reached. Obviously, the sensor demonstrated here is around 50 nm (even larger), which is about one order larger than that of the FSR in Ref. [26].

Finally, taking the sensor with full encapsulation as an example, the limit of detection (LOD) and figure of merit (FOM) of the sensor can be estimated as follows:

(9) $$\mathrm{LOD}=\frac{R_{\mathrm{OSA}}}{S_{\varepsilon }}=\frac{20\;\mathrm{pm}}{127.00\;\mathrm{pm}/\mu \varepsilon }=0.16\;\mu \varepsilon ,$$

(10) $$\mathrm{FOM}=\frac{S_{\varepsilon }}{\mathrm{FWHM}}=\frac{127.00\;\mathrm{pm}/\mu \varepsilon }{39.09\;\mathrm{nm}}\approx 3249,$$

in which *R_OSA_* is the resolution of the OSA used, *S_ε_* is the sensitivity of the sensor and FWHM is the full width at half maximum of the spectrum.

## 4. Discussion

### 4.1. Dependence of Sensitivity on Probing Wavelength

It can be seen from Equation (6) that the sensitivity will increase with the probing wavelength. In order to explore the dependence of the strain sensitivity on wavelength, the sensitivities corresponding to different wavelengths with the same sensor (during the same strain measurement) are investigated. These spectra are shown in Figure 3b,c. It is worth noting that because the full encapsulation will lead to a larger FSR, the number of dips will decrease significantly. However, too few peaks are not conducive to fitting. Thus, Figure 3d is not used.

**Figure 4:**
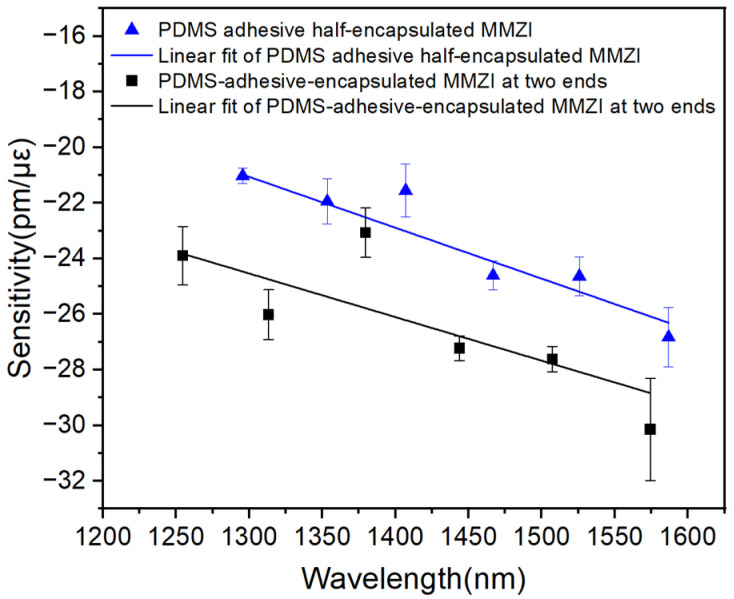
Dependence of strain sensitivity on wavelength.

For convenience of calculation, the wavelengths of the dips are listed in Table 1 and the dependences of sensitivity on wavelength are plotted in Figure 4. Results show that the absolute value of the sensitivity increases linearly with the wavelength. In addition, these two sensors show a very close slope, which indicates that their *P_e_* values are very close to each other.

### 4.2. Effect of Encapsulation Length on Sensitivity

**Figure 5:**
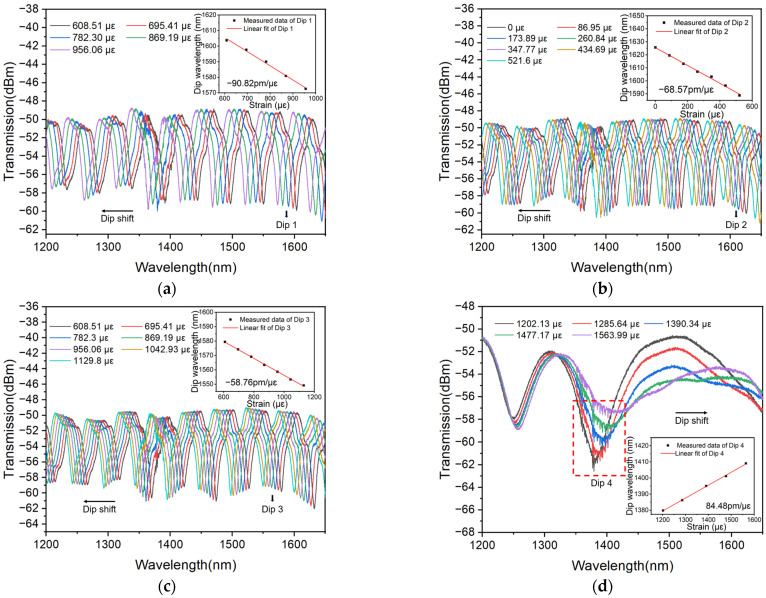
Spectrograms of the sensor strain sensitivity of PDMS with encapsulation lengths of (**a**) 1 cm on the left and right, (**b**) 1 cm on the left and 2 cm on the right, (**c**) 2 cm on the left and right and (**d**) 2.5 cm on the left and right (fully covered).

It can be seen from Section 3 that different encapsulation methods will lead to different sensitivities. Though the same encapsulation method is used, different sensitivities will be obtained. Taking the two-end encapsulation method for example, the MMZI is encapsulated by different lengths of PDMS to investigate the effect of the amount of polymer used on sensitivity. For ease of comparison, the same MMZI is used; however, a different amount of polymer is used in encapsulation, which is realized by adding the polymer successively to create encapsulations with different lengths. The lengths are listed in Table 2 in detail and the measured transmission spectra under different strains are shown in Figure 5a–d, respectively. As is shown, with the increasing length, the FSR first decreases and then increases, which is mainly because the increased amount of polymer will increase the strain. The increased stress pulls the sensor tighter and the length of waist region will increase. As a result, the FSR will decrease according to Equation (4).

However, when the whole waist region is covered by PDMS, the *n_eff_* will decrease dramatically due to the increasing refractive index of the surrounding (the surrounding is changed from air to PDMS) and the FSR will increase significantly. In addition, accompanying the changed FSR, the change in the sensitivity also shows the same trend (first decreases and then increases). In particular, when the MMZI is fully immersed in PDMS, a positive sensitivity is observed, which is mainly because when the waist region of the MMZI is covered by the polymer completely, the *P_e_* will be changed significantly, even changed from a negative value to a positive value. As a result, the dip shift and the sensitivity will also be opposite.

### 4.3. Effect of Polymer Used in Encapsulation on Sensitivity

**Figure 6:**
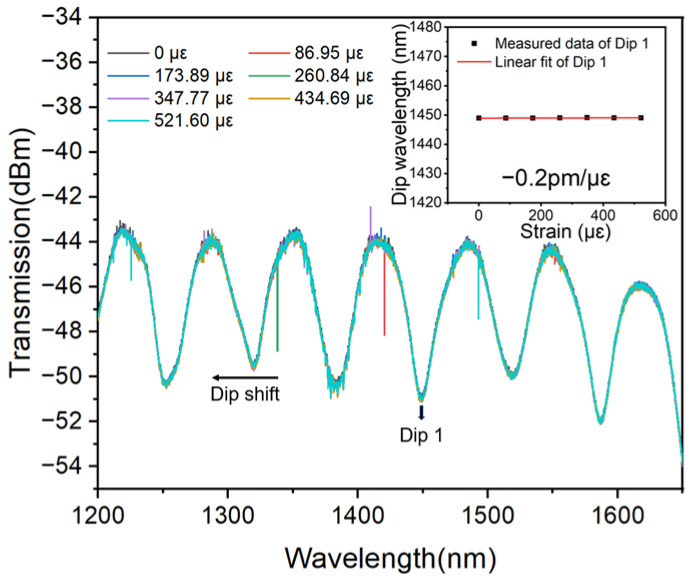
Strain response of the MMZI encapsulated by 302AB adhesive at two ends.

In the above encapsulation, PDMS is used. Actually, besides the PDMS, there are also many kinds of polymers, such as the 302AB adhesive with a Young’s module of 5 × 10^8^ Pa. In order to investigate the performance of the MMZI sensor encapsulated by different polymers, the sensors are also encapsulated at two ends using 302AB adhesive. The encapsulation and measuring methods are similar to those of sensors encapsulated by PDMS, only a different polymer is used in encapsulation. The experimental result is shown in Figure 6. It can be seen that the MMZI encapsulated by 302AB adhesive is basically insensitive to the strain with the negligible sensitivity of −0.2 pm/με, which is also in accordance with the fact that a polymer with a large Young’s modulus will resist the deformation induced by external strain, leading to low sensitivity. In other words, if one would like to develop a fiber sensor which is insensitive to strain, an adhesive with a large Young’s modulus can be used to encapsulate the sensor.

### 4.4. Dependence of Strain Sensitivity on Temperature

**Figure 7:**
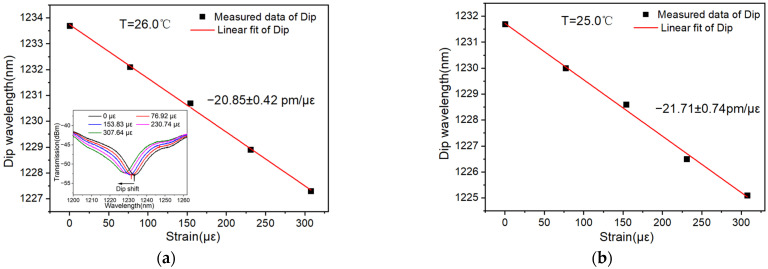

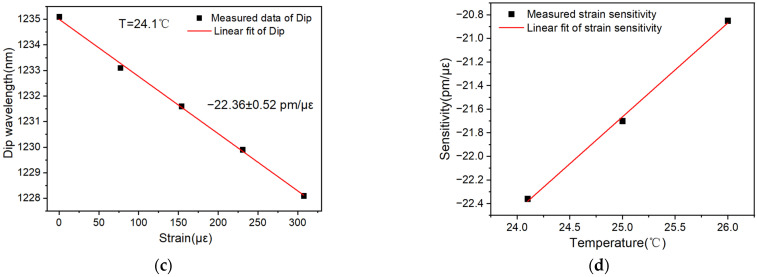
Strain sensitivity at room temperature of (**a**) 26.0 °C, (**b**) 25.0 °C and (**c**) 24.1 °C. (**d**) Dependence of strain sensitivity on temperature.

It is worth pointing out that all the above strain measurements are performed at room temperature. To provide more information regarding the dependence of sensitivity on temperature, strain sensitivity under different temperatures is tested. Considering that the temperature has varied from 24.1 °C to 26.0 °C in a single day recently, the strain sensitivity of the same sensor under three temperatures of 24.1 °C, 25.0 °C and 26.0 °C is tested with a two-end encapsulated MMZI sensor. The linear fitting of the typical dip is shown in Figure 7a with a sensitivity of around −20.85 pm/με (the dip shift of the sensor tested under 26.0 °C surroundings is shown in the inset of Figure 7a). With the same method, strain sensitivities under 25.0 °C and 24.1 °C can also be obtained, as shown in Figure 7b and Figure 7c, respectively. It can be seen that the influence of ambient temperature on strain sensitivity is small. By linear fitting in Figure 7d, it is found that there is a linear correlation between strain sensitivity and temperature. With the decreasing temperature, the strain sensitivity increases slightly.

### 4.5. Response Time

**Figure 8:**
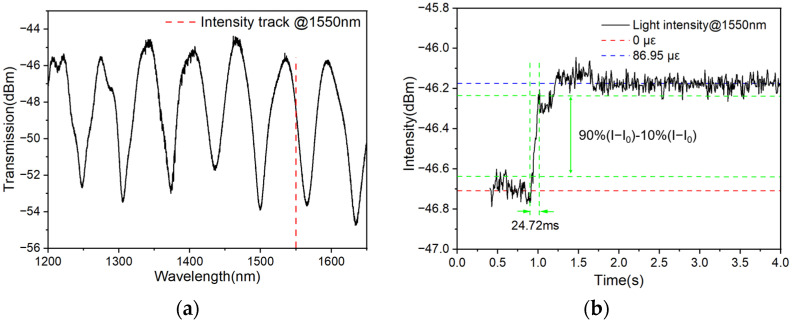
(**a**) Transmission spectrum of the strain sensor. (**b**) Typical temporal response of the output light under a 1550.0 nm wavelength.

For a practical sensor, response time is also one of the most important factors that should be considered in practical applications. To investigate the response time of the sensor, the sensor with 1 cm length encapsulation at two ends is tested experimentally, and its transmission spectrum is shown in Figure 8a. In the experiment, the intensity of the wavelength of 1550.0 nm is tracked. After introducing a sudden strain of 86.95 με on the sensor, the intensity of the wavelength changes accordingly, as shown in Figure 8b. Using the estimation method used in Ref. [30], the response time of the sensor can be estimated to be 24.72 ms.

### 4.6. Recoverability and Repeatability

**Figure 9:**
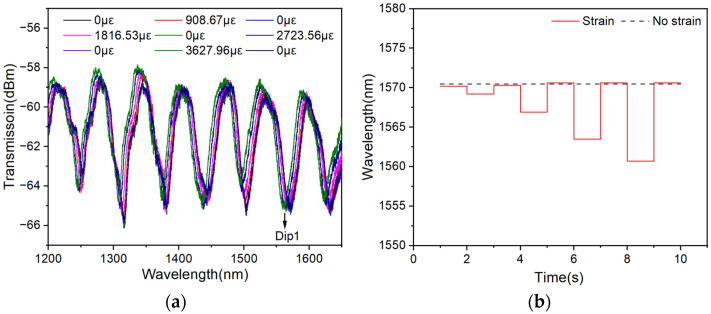
(**a**) Transmission spectra under different strains. (**b**) Wavelength of dip 1 under different strains.

Due to the low Young’s modulus of PDMS, good recoverability and repeatability of the sensor can also be guaranteed. To explore its recoverability and repeatability, different strains are applied and relaxed on the sensor randomly and repeatedly. The transmission spectra of the sensor during this process are shown in Figure 9a, and the wavelength of Dip 1 (1570.10 nm) is tracked and the changes in this dip are plotted in Figure 9b.

**Figure 10:**
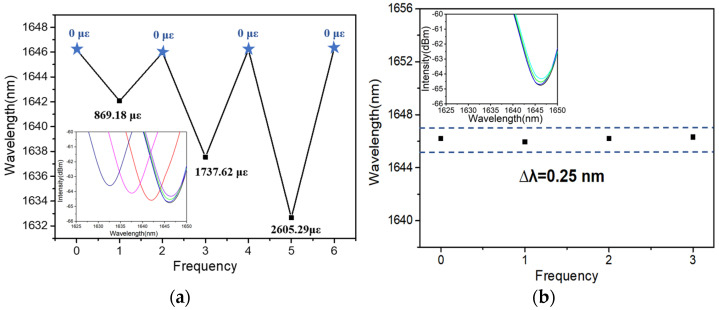
Wavelength fluctuation of (**a**) the typical dip in spectrum under different strains, and (**b**) the sensor under the free-strain state (0 με), recorded repeatedly.

To avoid occasionality, another MMZI is prepared for the test. Figure 10a records the wavelength of the typical dip in spectrum under different strains. To evaluate the dip shift induced by the repeated strain quantificationally, the wavelengths of the sensor under the free-strain state (0 με) are recorded repeatedly, as shown in Figure 10b. By comparing these wavelengths with the initial state (0 με), a deviation of around 0.25 nm is observed.

**Figure 11:**
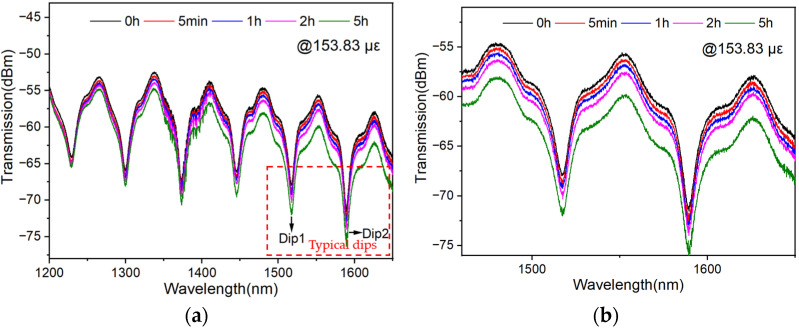
(**a**) Transmission spectra of MMZI at different times when strain was maintained at 153.83 με, (**b**) Amplification of transmission spectra of two dips.

In addition, to investigate the response of the sensor when stress is maintained, the peak shift of the sensor over a 5 h period is tracked when the strain is maintained at 153.83 με. The spectra are shown in Figure 11a. Two typical dips in the red box are selected for amplification, as shown in Figure 11b. Results show that the wavelength of each dip in the spectrum does not change significantly. Because the strain sensing is realized based on the dip tracking, the maintained stress will not affect the sensitivity significantly. However, it can also be seen that the light intensity may decrease, especially in the longer waveband, which may be induced by the increasing loss caused by fiber fatigue.

### 4.7. Comparison with Other Strain Optical Fiber Sensors

Finally, a comparison of the sensor demonstrated here with other kinds of strain sensors is shown in Table 3. It can be seen that compared with strain sensors based on MZI, FPI and hybrid strain sensors, the fabrication is much easier. Compared with strain sensors based on FBG, FPI and some hybrid strain sensors, the polymer-based microfiber sensor proposed here greatly improves the sensitivity of strain measurement. Compared with strain sensors based on MZI, FPI and some hybrid strain sensors, robustness is improved, befitting from the polymer encapsulation. In addition, the cost, fabrication and detection complexity of sensors are also summarized. For the cost of the sensor, the unit price of the fiber used is mainly considered. For the complexity of sensor preparation, we pay attention to the preparation process of each sensor for evaluation. In the evaluation of sensor detection complexity, the number of devices used in the whole detection system is focused on. It is obvious that the sensor demonstrated here shows the advantages of low cost, high sensitivity, simple fabrication, robust structure and simple detection.

## 5. Conclusions

In summary, a robust strain sensor based on microfiber is developed by combining an MMZI sensor with polymer encapsulation. By changing the encapsulation structure or the length of the polymer encapsulation, the sensitivity of this kind of sensor can be tuned from −20.95 pm/με to 127.00 pm/με, which is one or two orders of magnitude higher than that of a strain sensor based on a bare MMZI. Experimental results show that the sensitivity of the sensor will increase with the probing wavelength and the type of polymer material is also very critical to the sensitivity. In addition, the response time and the repeatability of the sensor are also evaluated experimentally. Results show that the response time of the sensor is on the level of tens of microseconds and the wavelength deviation before and after strain measurement is around 0.25 nm, which shows its good repeatability. Finally, the sensor demonstrated here is compared with existing strain optical fiber sensors. Evidently, the strain sensor based on the MMZI with PDMS encapsulation shows advantages of simple fabrication, robustness, high sensitivity and low cost, which shows great potential in strain sensing and measurement in the cases of monitoring the health of buildings and ships or earthquake monitoring.

## Figures and Tables

**Table 1:** Different sensitivities corresponding to different dips.

Encapsulations	Sensitivity *S*_ε_ (pm/με) and Central Wavelength *λ*_c_ (nm)						
	*S*_ε_/*λ*_c_	Dip 1	Dip 2	Dip 3	Dip 4	Dip 5	Dip 6
MMZI half-covered by PDMS	*S*_ε_	−20.95	−21.26	−22.43	−24.20	−24.20	−26.25
	*λ*_c_	1295.69	1353.40	1407.09	1466.71	1525.60	1586.86
MMZI encapsulated at two ends by PDMS	*S*_ε_	−23.26	−25.52	−22.98	−27.42	−28.07	−30.99
	*λ*_c_	1254.61	1313.47	1379.67	1443.73	1507.46	1574.44

**Table 2:** Comparison of strain sensitivity of the same MMZI with different encapsulation lengths.

Encapsulation Length (Left and Right)	Strain Sensitivity (pm/με)
1 cm, 1 cm	−90.82 ± 0.47
1 cm, 2 cm	−68.57 ± 0.62
2 cm, 2 cm	−63.04 ± 0.76
2.5 cm, 2.5 cm (Full-covered)	84.48 ± 1.32

**Table 3:** Comparison of different strain optical fiber sensors.

Sensor Structure		Sensitivity	Robustness	Cost	Fabrication	Detection Complexity	Year
FBG	FBG	0.90 pm/µε	High	Medium	Difficult	Simple	2018 [10]
MZI	MZI	−61.80 pm/µε	Low	High	Easy	Simple	2017 [16]
	MZI with MMF	−103.80 pm/µε	Medium	Medium	Easy	Simple	2018 [17]
	MZI	165.00 pm/µε	Medium	Low	Medium	Simple	2021 [19]
	MZI	−34.61 pm/µε	Low	Low	Easy	Simple	2022 [20]
	MMZI	−20.95~127.00 pm/µε	High	Low	Easy	Simple	This work
	MMZI	4.84 pm/µε	Low	Low	Easy	Simple	2014 [26]
FPI	FPI with PCF	31.58 pm/µε	Low	High	Difficult	Medium	2018 [21]
	FPI with SMF	1932.00 pm/µε	High	Low	Easy	Medium	2023 [22]
	FPI with HCF	3.29 pm/µε	Medium	Medium	Medium	Medium	2017 [31]
	FPI with HCF	8.62 pm/µε	Low	Medium	Difficult	Medium	2018 [32]
Hybrid	TCF and SMF	2.40 pm/µε	Medium	Medium	Easy	Medium	2022 [33]
	PCF and SMF	−71.92 pm/µε	Low	Medium	Medium	Simple	2023 [34]

## Data Availability

The original contributions presented in this study are included in the article; further inquiries can be directed to the corresponding authors.
